# Sensitization to Food and Inhalant Allergens in Relation to Atopic Diseases in Early Childhood: A Birth Cohort Study

**DOI:** 10.1371/journal.pone.0102809

**Published:** 2014-07-17

**Authors:** Chih-Yung Chiu, Yu-Lin Huang, Ming-Han Tsai, Yu-Ling Tu, Man-Chin Hua, Tsung-Chieh Yao, Kuo-Wei Yeh, Jing-Long Huang

**Affiliations:** 1 Department of Pediatrics, Chang Gung Memorial Hospital, Keelung, Taiwan; 2 Community Medicine Research Centre, Chang Gung Memorial Hospital, Keelung, Taiwan; 3 Division of Pediatric Pulmonology, Chang Gung Memorial Hospital, College of Medicine, Chang Gung University, Taoyuan, Taiwan; 4 Division of Allergy, Asthma, and Rheumatology, Department of Pediatrics, Chang Gung Memorial Hospital, and Chang Gung University College of Medicine, Taoyuan, Taiwan; The Hospital for Sick Children and The University of Toronto, Canada

## Abstract

**Objectives:**

A correct interpretation of sensitization to common allergens is critical in determining susceptibility to allergic diseases. The aim of this study was to investigate the patterns of sensitization to food and inhalant allergens, and their relation to the development of atopic diseases in early childhood.

**Methods:**

Children aged 0 through 4 years from a birth cohort in the Prediction of Allergies in Taiwanese Children (PATCH) study were enrolled. Specific IgE antibody against food and inhalant allergens were measured and their association between total serum IgE levels and atopic diseases were assessed.

**Results:**

A total of 182 children were regular followed up at clinics for a four-year follow-up period. The prevalence of food allergen sensitization increased markedly after 6 months of age, reaching up to 47% at 1.5 years of age and then declined significantly to 10% in parallel with a considerable increase in the prevalence of sensitization to inhalant allergens up to 25% at age 4. Food allergen sensitization appeared to be mainly associated with the elevation of serum total IgE levels before age 2. A combined sensitization to food and inhalant allergens had an additive effect on serum IgE levels after age 2, and was significantly associated with the risk of developing atopic diseases at age 4.

**Conclusions:**

Sensitization to food occurs early in life, in parallel with the rising prevalence of sensitization to inhalant allergens at older age. A combined sensitization to food and inhalant allergens not only has an additive increase in serum IgE antibody production but also increases the risk of developing allergic respiratory diseases in early childhood.

## Introduction

The prevalence of atopic diseases in childhood has significantly been increasing in the past few decades [1]–[3]. As the prevalence of atopic diseases in the population increases, early identification of atopic children is desirable. Potential predictors for atopic diseases in childhood or later in life have been studied widely [4], [5]. Early sensitization to allergens has consistently been identified as a risk factor for developing allergic respiratory diseases [6]. Assessment of allergen sensitization therefore is considered to be important in diagnosing and managing atopic diseases throughout childhood [7]. However, the interpretation of allergen sensitization and its clinical application in children sensitized to various allergens is challenging.

Allergic sensitization in infancy generally occurs first to food allergens. As children grow older, the majority of sensitization is directed against inhalant allergens [8]. Long-lasting sensitization to food allergens during the first two years of life has been regarded as a major risk factor for the development of allergic airway diseases [6]. In early childhood, the relationship between specific sensitization to food and inhalant allergens and their relevance to the occurrence of atopic diseases however has not been well determined.

Immunoglobulin E (IgE) is a critical component of allergic diseases. Although several studies have shown an association between the prevalence of atopic diseases and total serum immunoglobulin E (IgE) levels, total serum IgE levels are most useful in screening for atopic predisposition, rather than in the diagnosis or management of atopic diseases [9]–[11]. However, a moderate amount of specific IgE to a particular allergen may have much greater significance for a relatively lower total IgE levels [12]. Allergen-specific IgE antibodies also provide useful serological information in the differential diagnosis on IgE-mediated atopic diseases in young children with allergy-like symptoms [13]. Some persons with significant allergy problems can have normal, moderately or strongly elevated IgE levels. Although an elevated IgE level is associated with an increased risk of atopic diseases, the impact of sensitization to different allergens on the total serum IgE levels and the development of allergic diseases is still not well-defined.

The aim of this study was to determine sensitization to most common food and inhalant allergens in children aged 0 through 4 years from a birth cohort in the Prediction of Allergies in Taiwanese Children (PATCH) study. The patterns of sensitization to different allergens were assessed, and their relationship between total serum IgE levels and the development of atopic diseases were also examined.

## Materials and Methods

### Study Population

The Prediction of Allergies in Taiwanese Children (PATCH) study is a population-based birth cohort study initiated in 2007 to investigate the epidemiology and predictive factors of asthma and allergies in Taiwanese children. New born babies delivered at Chang Gung Memorial Hospital (CGMH), Keelung from October 1, 2007 to September 30, 2010 were recruited voluntarily and followed-up until the age of 4 years. Neonates born at more than 34 weeks of gestation with birth weight ≥2500 g were enrolled. Infants with any perinatal insult, significant neonatal respiratory difficulties, or congenital anomalies were excluded. Subjects who dropped out during the follow-up period were likewise excluded. This study was approved by the Ethic Committee of Chang Gung Memory Hospital (No. 100-0201B). Informed written consent was obtained from the parents of all study subjects.

### Data Collection

The parents of enrolled subjects were invited and underwent a standardized interview conducted by well-trained investigators for answering a questionnaire at birth and at 1, 2, 3 and 4 years of follow-up. The questionnaire was derived from the well-validated International Study of Asthma and Allergies in Childhood (ISAAC) questionnaire [14]. The details of information regarding demographic data, family atopy history and the health care of children including medical conditions were collected. Detailed questionnaire in regard to clinical symptoms and diagnosis of allergic diseases were also collected.

### Evaluation and Diagnosis of Atopic Diseases

The atopic diseases of childhood consist of atopic dermatitis, allergic rhinitis, and asthma. Information of current and past allergic symptoms for the diagnosis of atopic diseases was obtained from the validated ISAAC questionnaire [14]. Specific questions related to the development of allergic/atopic diseases and symptoms were also inquired and evaluated by a pediatric pulmonologist at outpatient clinics. Eczema was diagnosed as a pruritic rash over the face and/or extensors with a chronic relapsing course as described by Hanifin and Rajka [15]. Rhinitis was diagnosed as ever having the symptoms representing rhinitis such as sneezing, nasal congestion, itching, rhinorrhea or current use of medication for these symptoms [16]. Asthma was diagnosed as ever having asthma with the occurrence of wheeze or current use of asthma medication.

### Total and Allergen-Specific Serum Immunoglobulin E

Serum samples were collected and measured at 6 months, and 1, 1.5, 2, 3 and 4 years of age. The serum level of total IgE was measured by ImmunoCAP (Phadia, Uppsala, Sweden). Specific IgE was determined by a commercial assay for IgE (ImmunoCAP Phadiatop Infant; Phadia) against food and inhalant allergens. Egg and milk allergy are the most prevalent food allergies in infant and young Asian children [17]. In addition to egg and cow’s milk, wheat based supplementary food is most commonly supplied for young children in Taiwan. In contrast, *D. pteronyssinus* and *D. farina* are the two most common inhalant allergens causing sensitization in more than 95% of children in Taiwan [18]. In addition, molds are ubiquitously present in homes because of the warm and humid subtropical climate in Taiwan and *C. herbarum* is the most prevalent species [19]. Specific IgE antibody testing therefore included a mix of three most common food allergens (egg white, milk and wheat) and three most common inhalant allergens (*D. pteronyssinus*, *D. farina* and *C. herbarum*). The cut-off values for each ImmunoCAP Phadiatop Infant class 0, 1, 2, 3 and >3 are 0, 0.35, 0.7, 3.5 and ≥17.5 kU/L, respectively. Values of ImmunoCAP Phadiatop Infant of ≥0.35 kU/L (≥ class 1) were considered indicative of allergic sensitisation [10], [20].

### Statistical Analysis

Demographic data of population characteristics obtained by questionnaire and the prevalence of physician-diagnosed atopic diseases were collected and analysed. The Student *t* test was used to compare continuous variables, and the χ*^2^* or Fisher exact test was used to compare the nominal data. Differences between continuous variables with non-normal distribution were estimated with the Mann-Whitney test. The association of atopic diseases measured as a binary outcome with sensitization to food or inhalant allergens was calculated by the odds ratio (OR), using standard methods of logistic regression analysis. Statistical analysis was performed by using the Statistical Program for Social Sciences (IBM SPSS Statistics for Windows, Version 20.0. Armonk, NY: IBM) and graphs were drawn using GraphPad Prism Version 5.01 software (GraphPad Software Inc, California, USA). All statistical hypothesis tests were two tailed and a *P* value of less than 0.05 was considered to be significant.

## Results

### Population Characteristics

**Figure 1:**
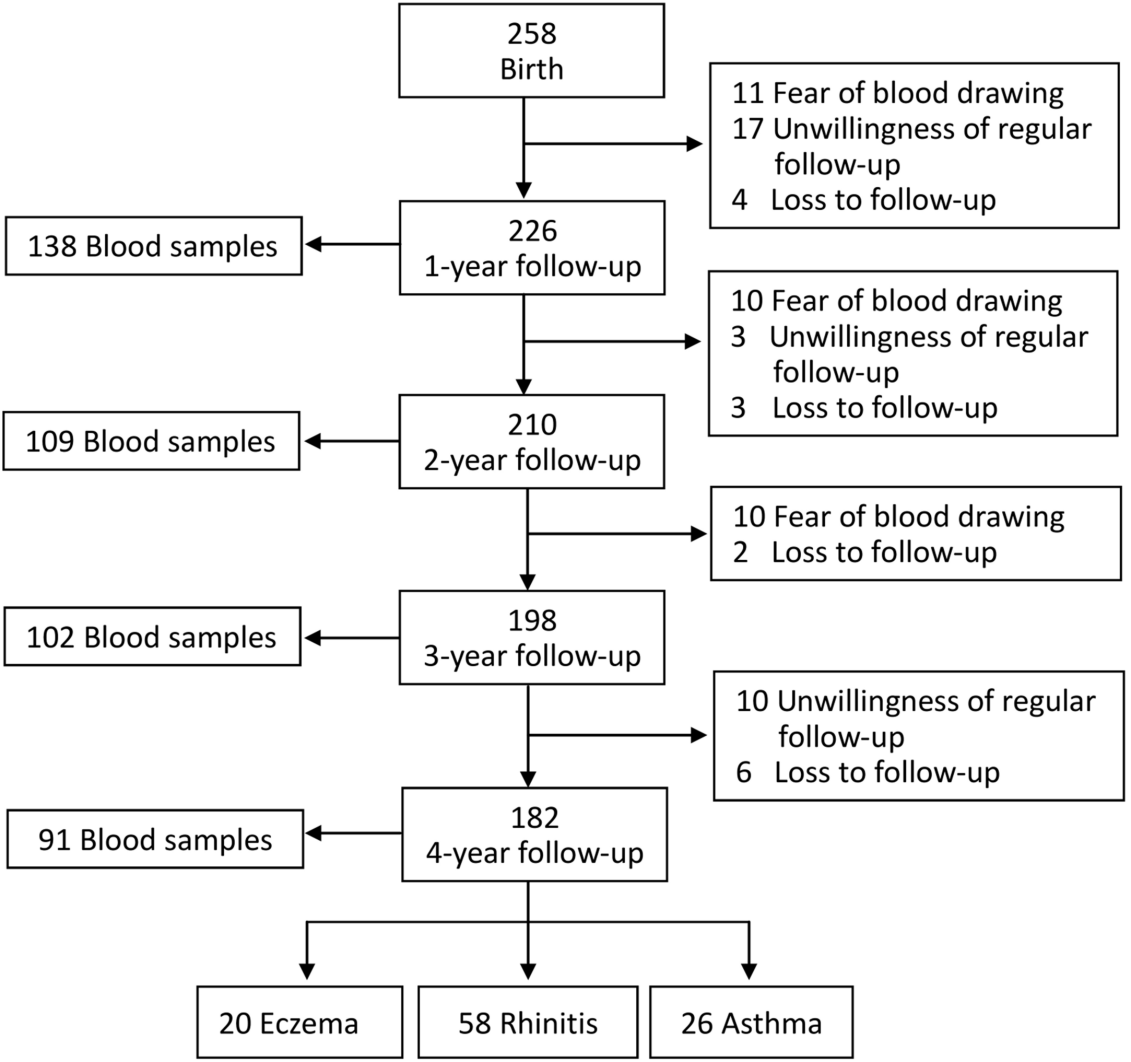
Schematic presentation of the recruitment process of the study subjects.

A total of 258 children who fulfilled the inclusion criteria were voluntarily enrolled; 226 (87.6%), 210 (81.4%), 198 (76.7%) and 182 (70.5%) children were regular followed up at clinics for a one, two, three and four year follow-up respectively. The major reasons for dropout were the fear of blood drawing and parents’ unwillingness of regular outpatient follow-up. Blood test for allergies was performed in 138 children at age 1, 109 children at age 2, 102 children at age 3 and 91 children at age 4. Atopic diseases including eczema, rhinitis and asthma were diagnosed in 20, 58 and 26 children, respectively, at 4 years of age (Fig. 1).

### Prevalence of Sensitization to Food and Inhalant Allergens

**Figure 2:**
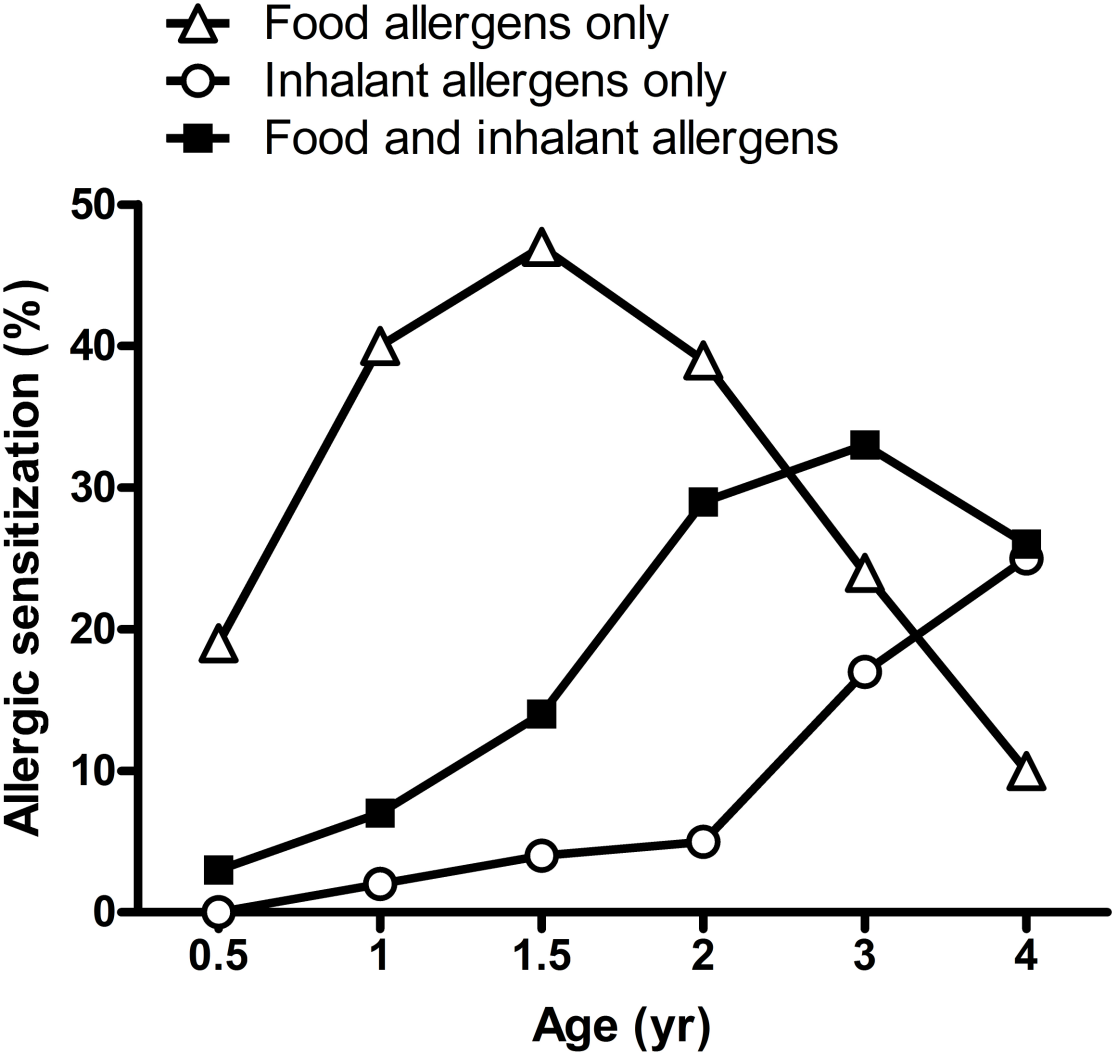
Sensitization patterns to food and inhalant allergens from age 0 to age 4.

Allergen-specific serum IgE for food and inhalant allergens was measured at 6 months, and 1, 1.5, 2, 3 and 4 years of age during follow-up. In food sensitization, the sensitization patterns of milk and egg white were similar. The prevalence of sensitization to milk and egg white allergens increased with increasing age until 1.5 years of age. By contrast, the prevalence of sensitization to wheat allergen was persistent low with approximately only 5%. Sensitization patterns to food and inhalant allergens are shown in **Fig.** 2. The prevalence of all food allergen sensitization increased markedly after 6 months of age and reached up to 47% at 1.5 years of age. In contrast, the prevalence of sensitization to inhalant allergens was only 5% at age 2, but increased markedly after 2 years of age. At 4 years of age, the prevalence of sensitization to food allergens declined significantly to 10% but there was a considerable increase in the prevalence of sensitization to inhalant allergens up to 25%. Furthermore, the prevalence of a combined sensitization to food and inhalant allergens showed to be increased gradually with increasing age, from 3% at 6-month-old to 26% at 4-year-old. Moreover, approximately fifty percent of the food-sensitized children at different ages had become sensitized to aeroallergens simultaneously at 4 years of age.

### Association between Food, Inhalant Allergen Sensitization and Serum IgE Levels

**Figure 3:**
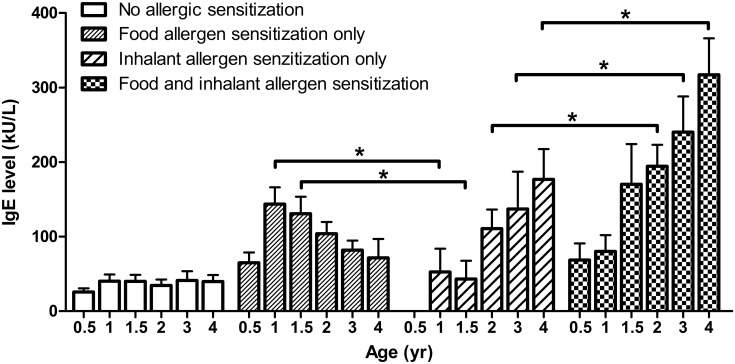
Changes of total serum IgE levels in children with sensitization to food and inhalant allergens at different years of age. Data shown are mean ± SEM. *P* values refer to the comparisons indicated by the marker. *P<0.05.

**Fig.** 3 shows the changes of total serum IgE levels in children with sensitization to food and inhalant allergens at different years of age. In children with food allergen sensitization, the total serum IgE levels appeared to be decreased with increasing age after age 1. In contrast, serum IgE levels showed to be increased gradually in children with inhalant allergen sensitization after age 2. In comparison to children with inhalant allergen sensitization, a significantly higher serum IgE level was found in children with food allergen sensitization at age 1 and 1.5. However, a statistically significant increase in serum IgE level was found in children with a combined sensitization to food and inhalant allergens with an addition effect after age 2.

### Association between Food, Inhalant Allergen Sensitization and Atopic Diseases

Table 1 presents the relationships between food, inhalant allergen sensitization and risk of atopic diseases by the age of 4 years. At 6 months of age, sensitization to food allergens, only egg white but not milk or wheat, showed a statistically significant risk in association with atopic diseases including eczema, rhinitis and asthma at age 4. In contrast, there was a significant increased risk for developing asthma and rhinitis but not eczema in children with sensitization to inhalant allergens after age 1 and age 2, respectively. A combined sensitization to food and inhalant allergens showed a significant risk in relation to all three atopic diseases before age 2 but it appeared to be more specific to rhinitis and asthma after age 2.

**Table 1:** Relationships between food allergen sensitization, inhalant allergen sensitization and risk of atopic diseases by the age of 4 years.

Age (yr)	Allergic sensitization	Eczema		Rhinitis		Asthma	
		OR	95% CI	OR	95% CI	OR	95% CI
0.5	Food allergens only	8.22	(1.72–39.51)*	5.29	(1.41–19.85)*	7.59	(1.75–33.00)*
	Inhalant allergens only	5.33	(0.45–63.22)	1.60	(0.14–18.29)	1.91	(0.11–32.01)
	Food and inhalant allergens	8.22	(0.67–101.03)	2.11	(0.18–24.37)	2.85	(0.17–48.86)
1	Food allergens only	3.00	(0.77–11.65)	1.16	(0.47–2.84)	3.00	(0.86–10.43)
	Inhalant allergens only	3.33	(0.59–18.72)	2.39	(0.58–9.93)	3.56	(0.71–17.79)
	Food and inhalant allergens	6.00	(0.88–40.87)	1.82	(0.39–8.51)	4.80	(0.74–31.08)
1.5	Food allergens only	1.00	(0.25–4.03)	1.82	(0.69–4.81)	9.00	(1.04–78.17)*
	Inhalant allergens only	7.60	(0.57–101.79)	5.18	(0.48–56.09)	38.0	(1.66–870.45)*
	Food and inhalant allergens	7.60	(1.07–54.09)*	8.64	(1.59–46.81)*	57.0	(4.36–744.71)*
2	Food allergens only	1.07	(0.23–5.05)	2.40	(0.81–7.14)	7.47	(0.82–68.10)
	Inhalant allergens only	4.00	(0.20–78.79)	8.00	(0.76–83.88)	48.0	(2.31–997.18)*
	Food and inhalant allergens	5.60	(1.15–27.37)*	8.40	(2.31–30.60)*	38.4	(3.95–373.09)*
3	Food allergens only	1.00	(0.15–6.85)	3.14	(0.85–11.67)	3.00	(0.40–22.30)
	Inhalant allergens only	3.50	(0.59–20.68)	4.50	(1.02–19.90)*	10.5	(1.50–73.67)*
	Food and inhalant allergens	3.50	(0.76–16.12)	6.29	(1.81–21.80)*	10.0	(1.71–58.63)*
4	Food allergens only	2.86	(0.63–12.92)	13.8	(1.18–161.71)*	34.5	(2.35–505.75)*
	Inhalant allergens only	4.38	(0.79–24.45)	4.22	(1.15–15.51)*	9.86	(1.61–60.24)*
	Food and inhalant allergens	6.13	(1.03–36.45)*	13.8	(3.40–55.96)**	13.8	(2.13–89.52)*

yr, year; OR, odds ratio; CI, confidence interval.

*P<0.05;

**P<0.001.

## Discussion

Atopic diseases are among the most common chronic disorders of childhood. In young children, the diagnosis of atopic diseases is mainly based on clinical evaluation. Although assessment of allergen-specific IgE antibodies provides helpful information to the clinician, a correct interpretation of sensitization to common allergens is critical in determining susceptibility to allergic diseases. In this study, the prevalence of sensitization to food allergens appeared to occur and increase in early infancy as in previous publication [6]. However, it declined significantly after age 2 and there was concomitantly a compensatory increase in the prevalence of sensitization to inhalant allergens [21]. Most importantly, the prevalence of a combined sensitization to food and inhalant allergens showed to be increased with increasing age. These findings indicate that changes in sensitization to food and inhalant allergens varies with age in early childhood, which may play an important role in allergic response and the development of atopic disorders.

An allergic reaction occurs when the immune system overreacts to the allergen by producing IgE antibodies. IgE is a type of antibody that is present in minute amounts in the body but plays a major role in allergic diseases [22]. In this study, the changes of serum IgE levels were consistent with the sensitization patterns to food and inhalant allergens in relation to age. A high prevalence of sensitization to food allergens and a significantly elevated IgE levels in children with food allergen sensitization at age 1 and 1.5 indicates that food allergen sensitization may mainly be associated with the elevation of serum IgE levels before the age of 2. With the decline in the IgE levels induced by food allergen sensitization, the prevalence of sensitization to inhalant allergens increased after age 2 and an additive increase in serum IgE levels was in parallel with an increase in the prevalence of a combined sensitization to food and inhalant allergens, indicating that sensitization to inhalant allergens may play an important role in allergic responses after age 2. Clinically, total serum IgE levels have shown to be associated with the severity of allergies among young children [23]. In this study, the total serum IgE values was strongly associated with the striking cosensitization between food and inhalant allergens, suggesting that increased IgE levels induced by multiple allergen sensitizations may contribute to a severe allergic response and increase a risk for developing atopic diseases [24].

Sensitization to allergens has been recognized as the most important risk factor for atopic diseases. Many studies have suggested that the occurrence and severity of symptoms in later childhood are directly related to allergen sensitization in infancy [6], [25]. Sensitization to food allergens is more common in early life and has been thought to be a significant factor for infantile eczema as in this study [6], [26]. However, approximately half of young infants with food allergen sensitization were more likely to be allergic to inhalant allergens, and were at increased risk for developing respiratory allergic diseases by the age of 4 years. Furthermore, egg sensitization in infancy was associated the most strongly with the development of atopic diseases. These findings support the previous report that the presence of egg-specific IgE antibodies constitutes the earliest marker of atopy [21]. Filaggrin gene defects are strongly associated with atopic eczema [27]. Furthermore, the increased skin permeability may increase the risk of sensitization to food and other allergens, and the risk of developing atopic diseases [28]. An infant with a genetic predisposition has been reported to develop antibodies to foods at a very early age, mostly to egg white and milk [29]. Although it has been proposed that exposure to egg proteins occurs via mother’s milk due to the lack of egg white in the young infant’s diet [30], a genetic susceptibility to allergy may also potentially play an important role in such instances.

The environmental factor has been studied most extensively as a potential risk for allergic sensitization and manifestation of atopy. In sensitized children, early exposure to inhalant allergens not only determines the risk of asthma, but also the time of the onset of the disease [31]. In this study, sensitization to inhalant allergens mainly occurred after age 2 and appeared to be more specific to the development of asthma. This finding may explain why the majority of school-aged asthmatic children have their onset of asthma during the first 2 to 3 years of life [32], [33]. Furthermore, food allergen sensitization in infancy not only increased the risk of sensitization to inhalant allergens but also the risk for developing atopic diseases at older age. It must be emphasized that a well recognition and interpretation of the changes in sensitization to food and inhalant allergens in early life may help clinician to predict the occurrence of atopic diseases and determine possible prevention strategies in clinical practice.

Limitations of this study include a relatively small sample size of only 182 case subjects and a limited power to detect a statistically significant association for subanalyses. A 4-year follow-up in this birth cohort study may not predict whether children with allergic sensitization will develop atopic diseases in later years. The strength of the present study lies in its longitudinal design, allowing sequential and concurrent measurements of serum total IgE, allergen-specific IgE for allergic sensitization and the accurate diagnostic evaluations for atopic diseases at outpatient clinics. Although a larger and longer study may be needed to confirm the findings presented in this study, this birth cohort study has demonstrated the importance of allergic sensitization patterns in developing atopic diseases in early childhood.

In conclusion, sensitization to food is mostly common in early life, in parallel with the rising prevalence of sensitization to inhalant allergens at older age. Serum total IgE levels appear to be associated with the response to different types of allergens. Sensitization to food, especially egg white is highly related to eczema, whereas sensitization to inhalant allergens appears to be more specific to the development of rhinitis and asthma. A combined sensitization to food and inhalant allergens not only has an additive effect on serum IgE antibody production but also increases the risk of developing atopic diseases. A fully understanding of the prevalence of allergen sensitization in different age would help early diagnosis and intervention of atopic diseases in early childhood.
